# Risk for intracranial pressure increase related to enclosed air in post-craniotomy patients during air ambulance transport: a retrospective cohort study with simulation

**DOI:** 10.1186/s13049-017-0394-9

**Published:** 2017-05-12

**Authors:** Helge Brändström, Anna Sundelin, Daniela Hoseason, Nina Sundström, Richard Birgander, Göran Johansson, Ola Winsö, Lars-Owe Koskinen, Michael Haney

**Affiliations:** 1 Anesthesiology and Intensive Care Medicine, Umeå University, Umeå, Sweden; 2 Biomedical Engineering, Medical Radiation Sciences, Umeå University, Umeå, Sweden; 3 Radiology, Medical Radiation Sciences, Umeå University, Umeå, Sweden; 4 Neurosurgery, Pharmacology and Clinical Neurosciences, Umeå University, Umeå, Sweden

**Keywords:** Air ambulance, Pneumocephalus, Intracranial pressure

## Abstract

**Background:**

Post-craniotomy intracranial air can be present in patients scheduled for air ambulance transport to their home hospital. We aimed to assess risk for in-flight intracranial pressure (ICP) increases related to observed intracranial air volumes, hypothetical sea level pre-transport ICP, and different potential flight levels and cabin pressures.

**Methods:**

A cohort of consecutive subdural hematoma evacuation patients from one University Medical Centre was assessed with post-operative intracranial air volume measurements by computed tomography. Intracranial pressure changes related to estimated intracranial air volume effects of changing atmospheric pressure (simulating flight and cabin pressure changes up to 8000 ft) were simulated using an established model for intracranial pressure and volume relations.

**Results:**

Approximately one third of the cohort had post-operative intracranial air. Of these, approximately one third had intracranial air volumes less than 11 ml. The simulation estimated that the expected changes in intracranial pressure during ‘flight’ would not result in intracranial hypertension. For intracranial air volumes above 11 ml, the simulation suggested that it was possible that intracranial hypertension could develop ‘inflight’ related to cabin pressure drop. Depending on the pre-flight intracranial pressure and air volume, this could occur quite early during the assent phase in the flight profile.

**﻿Discussion:**

﻿These findings support the idea that there should be radiographic verification of the presence or absence of intracranial air after craniotomy for patients planned for long distance air transport.

**Conclusions:**

Very small amounts of air are clinically inconsequential. Otherwise, air transport with maintained ground-level cabin pressure should be a priority for these patients.

## Background

Air that occurs in body spaces that normally do not contain air, or cannot eliminate the air directly by some form of ventilation, normally will be resorbed over time and disappear. If intracranial air is present post-operatively [1, 2], for example after the surgical evacuation of an intracranial expansive lesion, it will also be resorbed over time, over weeks [3, 4]. In modern, centralized, specialty care, patients receiving neurosurgical operative interventions (commonly borr hole or mini-craniotomy) at a tertiary care hospital may be transported back to their home hospital early after their operation accompanied by critical care personnel [5, 6], where post-operative intracranial air (if present) has not yet been resorbed. This study concerns assessment of risk for adverse medical consequences related to expansion of post-operative intracranial air. This is a clinical risk for patients who are transported post-operatively between hospitals by fixed wing air ambulance, where cabin pressures change in-flight.

When inside the cabin of an aircraft gaining altitude with the cabin pressure valve in the usual operating position, cabin pressure will decrease down to 0.74 atm, and air in closed body spaces will expand according to Boyle’s ‘law’ (a fixed relationship between gas volume, pressure, and temperature) [7]. In theory, if the flight altitude change occurs more slowly, and likewise the cabin pressure change also occurs more slowly, then it is thought that ICP changes might not be as dramatic, related to cerebrospinal fluid flow out of the cranium over time [8].

The intracranial volume-pressure relationship shows that at higher levels of ICP intracranial compliance decreases dramatically [9], meaning that small changes in intracranial air volume can potentially bring about clinically important changes in ICP. When ICP levels increase above capillary pressures, compromise of cerebral perfusion can occur.

It is not known at which amount of pneumocephalus and at which starting ICP there would develop injurious ICP elevations when patients are exposed to reduced ambient atmospheric pressure. Patients with ICP at the high limits of normal, and with large amounts of intracranial air on the ground and before air transport, would clearly be at risk [10–17]. Patients with low ICP and very small amounts of intracranial air on the ground before air transport can be assumed to be at low risk. There is much uncertainty about patients with moderate ICP at rest and moderate amounts of pneumocephalus. An additional complexity in this clinical assessment is that it is not clinically feasible to measure resting ICP in patients who do not have an implanted intracranial manometer or fluid-filled catheter for this purpose. Consequently, resting ICP in this setting must be estimated based on indirect means.

With commercial flights, where patients sometimes are transported over long distances, there are standard flight profiles which include efficient cruising altitudes, and also reduced cabin pressures during flight [18–20]. Air ambulances with pressurized cabins can offer the possibility of pressurizing the cabin to approximately sea level, provided that they can be allowed a flight profile with quite low and inefficient cruising altitudes. If a clinician knew with confidence how much effect the lower atmospheric pressure in an air ambulance cabin at cruising level would have on ICP, then they could make an informed decision and chose a special ‘ambulance’ flight profile, with cabin pressure at sea level. Alternatively, since flying longer distances with sea-level cabin pressures is more costly, slower, and often more turbulent, this should be avoided if not indicated for patient (or crew) safety.

The first specific hypothesis was that intracranial air was common in the immediate post-operative days after evacuation of subdural hematoma. The second hypothesis was that combinations of resting ICP and environmental (cabin) pressure with corresponding intracranial air volume increases can be identified which define ranges, below which high ICP would not be expected during air transport. Also, conversely, ranges of pre-transport ICP and intracranial air volume could be identified, above which high ICP would be expected during air transport. We aimed to test this in connection with a cohort of post-craniectomy patients who in our region are frequently transferred by air ambulances from the tertiary hospital back to their distant referring hospital. We also include analysis related to cabin pressures typical of long distance air ambulance transfers where there is no option for maintaining sea level cabin pressure. We aimed to test these hypotheses in a regional cohort of subdural hematoma evacuation patients. An established model for estimating ICP changes was used, which incorporates starting ICP and intracranial air volume inputs to transform observed patient intracranial air volumes to an expected ICP during a theoretical flight and cabin pressure change.

## Methods

### Study design

This was a retrospective observational cohort analysis, which began with post-operative intracranial air volume observations. These were used to generate estimates for intracranial air volume expansion and ICP at different aircraft cabin pressures and rate of change of cabin altitude in flight. An established model [8] for nonlinear transformation of air volume expansion into intracranial pressure was applied.

### Subjects

The medical records and radiographic images were identified and examined for consecutive subdural hematoma with borr hole or mini-craniotomy and hematoma evacuation patients admitted to the regional tertiary hospital (University Hospital of Umeå, Sweden) during the calendar year 2014.

### Measurements

Pre-operative and last (before discharge or transfer) postoperative computed tomographic (CT) image of the head for this cohort were identified. All scans were performed as spirals with 3 mm reconstructions in axial (HyFa-line), coronal (parallel to dorsal brainstem) and sagittal orientations. Volume calculation of subdural hematomas, as well as subdural air collections and epidural hematomas, was performed. Ellipsoid approximation was used, V = r1* r2* r3*4/3*π, where r was defined as the longest continuous diameter in the three perpendicular directions divided by 2. The three diameters were defined as follows: first, parallel to the calvarium in the axial plane; second, parallel to the calvarium in the coronal plane; and third, perpendicular to the calvarium, most commonly in the coronal plane. If the collection was located anteriorly or posteriorly, the axial or sagittal plane was used. When collections were crescent shaped, the diameter was divided into a number of continuous parts (2 to 7), not intersecting the adjacent parenchyma. All radiological evaluations and volume calculations were performed by a single experienced neuroradiologist.

There was no measurement of post-operative ICP at the time of the CT demonstrating intracranial air.

### The model for transformation of starting ICP and air volume to final ICP result

**Fig. 1:**
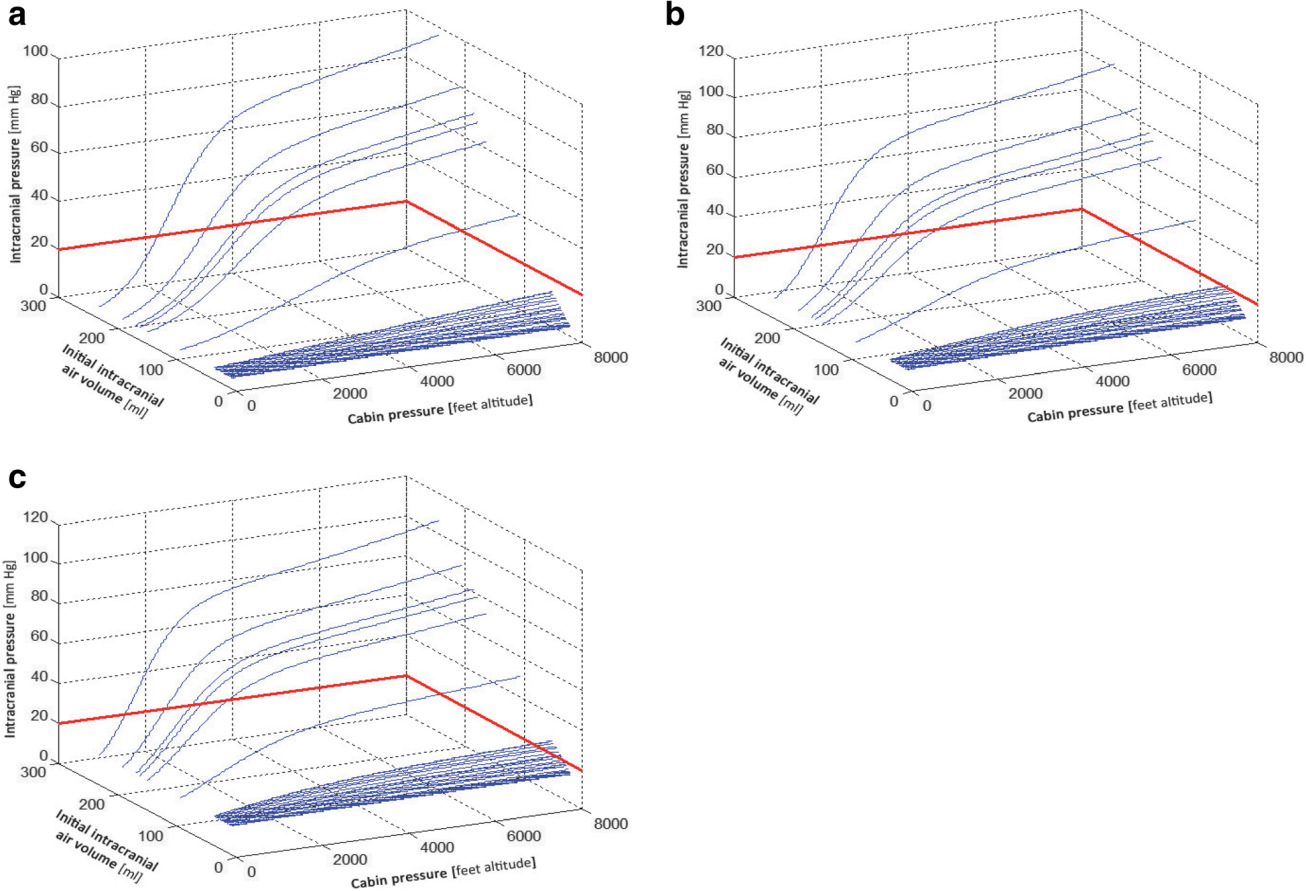
Observed intracranial air volumes and simulated ICP changes. These represent the observed post-surgery intracranial air volumes, with simulated decrease in cabin pressure (to 0.74 atm or 8000 ft altitude) during flight ascent. In (**a**), starting ICP is 5 mmHg. A clinical treatment target of ICP under 20 mmHg is shown as the red line on the ICP axis. Even with low starting ICP, one can note that with larger starting IC air volumes, ICP is expected to rise quickly even during early flight phases (climbing to cruising altitude). In (**b**), the starting is ICP 10 mmHg. It is notable that in this model, when there are larger amounts of air, and with ‘ascending’ altitude, that the estimated intracranial pressure rises quite quickly, within the first 3000 ft ascent. In (**c**), the starting ICP is 15 mmHg. With higher starting ICP, even subjects with small amounts of intracranial air volume can have air expansion and intracranial pressure increases well above a safe level of 20 mmHg

During decrease in ambient external pressure (as in an aircraft cabin during ascent in flight) and given unchanged temperature, intracranial gas volume will increase. As intracranial gas volume increases, this will also lead to an increase in ICP, as long as the dura mater and/or calvarium is intact. The model [20] is used to simulate ICP and intracranial air volumes during ascent from sea level (1 ATM) to 8000 ft. (0.74 ATM). The parameters used for ICP estimation at different air volumes were as follows: cabin altitude rate of change = 2.54 m/s (500 ft/min), outflow resistance of the cerebrospinal fluid system = 1.29e^+11^ Pa/(m^3^ s) and pressure volume index (PVI) of the cerebrospinal fluid system = 12.6 ml. The highest level (8000 ft altitude) (see Fig. 1) represents the cabin pressure at cruising altitude in a typical long haul commercial jet, as well as most executive or business aircrafts with pressurized cabins, flying at the most efficient cruising altitude. The relatively higher ‘cabin pressures’ represent levels that can be achieved if these aircrafts choose to fly at lower altitudes, with intentional increases in cabin pressure. Starting ICPs for the simulations were chosen as low/normal (5 mmHg), normal (10 mmHg), and high/normal (15 mmHg).

### Analysis

Descriptive statistics were used to describe the relative frequency and amounts of post-operative intracranial air in the subdural hematoma cohort. For the different simulated air ambulance cabin pressures, ranges including representative point intervals for starting ICP and intracranial air volumes were chosen to include the observed start intracranial air volumes in the post-craniectomy cohort.

## Results

There were 119 patients included in the study, with demographics shown in Table 1. In all these, the SDH was treated operatively with craniectomy (burr hole) or mini-craniotomy and hematoma evacuation. Subdural passive drains are standard during 12–24 h postoperatively. All of these patients received their operation in the same Department. They were all anesthetized using the same neuroanesthesia practice routines, which include using propofol, remifentanil and sevoflurane as anesthetics. No nitrous oxide was administered to these patients. The surgical management after the operation was to close the scalp incision to prevent leakage (or air suction), and the incision area was covered with a bandage, minimizing the risk for any air passage across the incision. The patient was treated without head elevation until the subdural drains were removed and a suture applied to close the skin stoma.

**Table 1:** Cohort demographics

	*n* =	Age (years)	Age range (years)
Total	119	76 ± 9	40–98
Men	95	75 ± 10	40–98
Women	24	79 ± 7	65–92
Lowest RLS after operation, for patients (*n* = 34) that had pneumocephalus			
	Pre-op	Last post-op	
RLS 1	12	17	
RLS 2	16	13	
RLS 3	3	0	
RLS 4	0	0	
RLS 5	0	0	
RLS 6	1	0	
RLS 7	0	0	
RLS 8	0	0	
Missing values	2	4	

Mean values ± standard deviation presented. RLS: Reaction level scale. RLS 1: Awake and oriented. RLS2: Drowsy or confused, response to light stimulation. RLS3: Very drowsy or confused, responsive to strong stimulation. RLS4 Unconscious, localizes but does not ward pain. RLS 5: Unconscious, withdrawing movement on pain stimulation. RLS 6: Unconscious, stereotypical flex movements on pain stimulation. RLS 7: Unconscious, stereotypical extension movements on pain stimulation. RLS 8: No response to painful stimulation

Of the 34 patients that had intracranial air post-operatively, 30 required no post-operative ventilator support. The remaining 4 were transferred from the operating room to the intensive care unit and briefly received ventilator support, though all were discharged from the intensive care within 1 day. These 4 had their post-operative head CT done while on the ventilator. All patients were treated initially after their operation with supplement oxygen, titrated to an oxyhemoglobin saturation of 92–96%.

Review of the post-operative head CT images showed that of these 119, 108 had at least one post-operative head CT examination. Fifty of the 108 had more than one post-operative head CT, and for these, the last CT image was the one included in the analysis. Of the 108 subjects, 34 had post-operative intracranial air on the head CT. Where there were multiple post-operative head CT studies done for an individual patient, and where there was intracranial air detected, it was the study with the largest intracranial air volume that was used in the analysis.

Air volume on the post-operative head CT was derived from the multiplane reconstructions. From these estimations, 28/34 had intracranial air volumes less than 40 ml total (range 6.5–37.2 ml), and 6/34 had intracranial air volume more than 90 ml (range 93.9–230.9 ml) (Table 2).

**Table 2:**
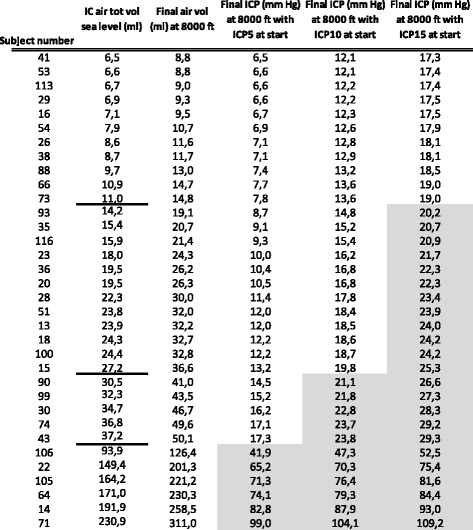
Total post-operative intracranial air volumes, intracranial pressure at sea level and estimated at final cruising altitude

Final air volume and final ICP refers to those estimated for cabin altitude 8000 feet in ascending order for amount intracranial air. Shaded areas show where intracranial pressure (ICP) would be expected to increase over 20 mm Hg at cruising altitude (cabin pressure = 8000 ft).Abbreviations: Intracranial (IC), total (tot), volume (vol), calculated (Calc), starting intracranial pressure at sea level 5 mm Hg (ICP5), starting intracranial pressure at sea level 10 mm Hg (ICP10), starting intracranial pressure at sea level 15 mm Hg (ICP15)

Table 2 presents IC air volumes observed on CT in ascending order. The final air volume estimation at ‘8000 ft. altitude’ is presented with each initial volume observation. Then, the simulation results for final ICP estimation are presented for each of the 3 hypothetical starting ICPs and the observed starting IC air volume. Resulting ICPs at 8000 ft. altitude estimates show that ICP is expected to remain under 20 mmHg for all IC volumes 11 ml or lower, and this includes those with hypothetical starting ICP 15 mmHg. For the ICP 5 mmHg at start, the final ICP at 8000 ft. was estimated to exceed 20 mmHg for those starting with IC air volumes (sea level) over approximately 40 ml air (Fig. 1). For the ICP 10 mmHg at start, the final ICP at 8000 ft. was estimated to exceed 20 mmHg for those starting with IC air volumes (sea level) over approximately 27 ml air. For the simulation with starting ICP 15 mmHg, ICP was expected to exceed 20 mmHg for those with starting IC air volume (sea level) of only 14 ml.

Continuous estimations of ICP resulting from the different observed starting IC air volumes along with different starting ICP levels demonstrate the ranges of the potential ICP events related cabin pressure change (Fig. 1). Intracranial air volumes increase as cabin pressure decreases, and these are shown in the third column of Table 1, as well as in Fig. 1. The model demonstrates relatively minor changes in IC air volumes which accompany quite large ICP changes during simulated cabin pressure decrease. As an example, when the subject with 37.2 ml IC air (sea level) goes to simulated cabin pressure 8000 ft., the air volume is expected to increase to 50.1 ml, or approximately 25%. At the same time, if starting ICP was on the higher side (15 mmHg) at start, and closer to the less compliant portion of the intracranial pressure-volume relationship, then the expected ICP at 8000 ft. would be 29.3 (or approximately 50% increase). In addition, the model shows (Figures) that there is a relatively steep increase in ICP in the first ‘ascent’ phase.

## Discussion

The main findings include first, the majority of post-op patients in this typical clinical post-surgery cohort did not have intracranial air post-operatively (CT exam), but approximately 1/3 did have some IC air. These findings confirm that some post-surgery subdural hematoma patients have intracranial air post-operatively [3, 13, 15].

The vast majority of post-operative patients in this cohort had little or no intracranial air, where a clinician could reasonably conclude that it would be safe for them to fly at usual cabin pressurisation with no risk for complication related to air volume expansion and ICP increase. This clinical decision, however, would need to be made based on a post-operative (or pre-transport) assessment of intracranial operative results including air volume (easily calculated from a 3 dimensional CT reconstruction). Of those 34/108 in this cohort that had IC air (32%), for 23/108 (21%), there was enough postoperative IC air that it potentially would have been a threat had the patient been transported in an air ambulance with usual cabin pressure. Further 11/108 (10%) had small enough IC air volumes (11 ml or less) for the simulations to show that even at the final cruising altitude with cabin pressure at 0.74 atm (8000 ft. altitude), ICP would not rise above 20 mmHg if the ICP was 15 mmHg or lower at the start of the flight.

These findings suggest that there is enough likelihood of post-operative IC air that one cannot presume that there is none. Nor, can one presume that just time passing before flying will resolve post-operative intracranial air predictably, since this is recognized to require weeks to resolve spontaneously [13].

A second major finding was that, according to the simulations, many of the subjects with more than 11 ml of post-operative intracranial air (observed sea level IC air volumes ranging from 14 to 230 ml) would be exposed to dangerous increases in ICP during flight ascent. If air transport was necessary at that time, these subjects would have benefited from physician awareness of patient IC air, and the likelihood of ICP increase given a normal flight profile. Either an adjustment in the flight profile and cabin pressure would be needed, or postponement of the patient air transfer.

At what point dangerous ICP levels are reached during flight ascent including IC air also depends on the starting ICP at sea level or ground level. Of course, ICP is not static, and varies with activity. The starting ICP levels in the simulations were chosen to represent average levels which were low, moderate and high normal. For patients after subdural hematoma evacuation, it is reasonable to assume that within a typical cohort, some would fall into each of these ranges, based on residual and resolving tissue edema or/and some space-occupying residual hematoma. When the resting, ground level ICP is relatively high, there is less margin for increase (with IC air expansion) before dangerous ICP levels are reached (Fig. 1). For the low ICP starting level, only those with IC volumes over 100 ml (6/34) had ICP increases to dangerous levels during flight ascent and cabin pressure decrease. Starting ICP matters, but it is not something that is easily measured at this time clinically in post-operative patients without invasive monitors. This is why this type of advanced model is needed to simulate ICP changes related to IC air expansion in a changing environment.

A third finding was that for subjects with more intracranial air, or if starting at higher sea level ICP, the increase in ICP during flight level ascent and cabin pressure decrease occurred relative rapidly (Fig. 1). Thereby, signs of ICP increase probably would be detectable early in the ascent phase of the flight plan for patients with either large volumes of IC air, a high starting ICP, or both. This has a clinical implication that where IC air and higher ICP might be suspected (but not confirmed), medical air ambulance personnel should be aware that dramatic ICP increases may occur very early in the flight plan ascent. It is not only cruising altitudes and corresponding cabin pressure decreased levels which are dangerous for these patients.

Clinical implications from these findings are several. First, in patients with intra-cranial surgery that need to be transferred over distances requiring air ambulance involvement, clinicians should recognize that IC air can be present in unpredictable amounts. Only a post-operative head CT scan, close in time to the transport date, can identify this. Neuroradiologists can easily approximate air volumes from head CT studies. Without this diagnostic information, it would be prudent to assume that there is IC air since it is not uncommon in a clinical cohort like the one in this study. Assuming that there is IC air which is over a minimal amount (11 ml in this study) means that transporting patients with sea-level air pressure in the cabin is safe. Still, if a post-operative head CT can show that there is air but only a minimal amount (11 ml or less), then these findings suggest that this air by itself, along with a routine flight plan and normal cabin pressure, should not be a danger to this patient. Conversely, most patients would be expected to need sea level cabin pressure for air ambulance flights.

For some transports, for patient travel or repatriation over long distance, private air ambulance where cabin pressure can be regulated may not be a readily available option. Commercial air travel may be preferred, even for patient transport with health care personnel. One must recognize that cabin air pressure in commercial long haul aircraft is normally between 6000 and 8000 ft. depending on the airframe type and age, though cabin pressures in commercial passenger airframes have been reported to be as ‘high’ as 9000 ft [18, 19]. It is prudent to confirm the absence of intracranial air in any post-craniotomy (recent) patient scheduled for air transport on a commercial aircraft, rather than risk an unplanned diversion of a jumbo-jet due to symptoms developing during ascent related to reasonably anticipated IC air expansion.

Sea level cabin pressure is indicated in an air ambulance transport setting when the medical crew has any suspicion that IC air can lead to a change or worsening in a patient’s neurological condition. This includes the situation where there is suspicion of IC air, but where this has not been quantified as in patients that are not awake, and when changes in neurological status or condition may be difficult to detect.

## Conclusions

In the absence of clear clinical information about post-operative IC air, medical crews must assume that a post craniectomy/craniotomy patient can have IC air that will expand during air transport with usual cabin pressurisation. Multiple combinations of starting ICP and IC air volumes lead to ICP over 20 mmHg as ‘cabin pressure’ decrease as it would in flight. It is important to perform a post-operative head CT and estimate IC air volumes for post-intracranial surgery patients if there is a possibility that they will be transported with an air ambulance.
